# The Construct of Medical and Non-Medical Marijuana—Critical Review

**DOI:** 10.3390/ijerph19052769

**Published:** 2022-02-27

**Authors:** Andrzej Silczuk, Daria Smułek, Marcin Kołodziej, Julia Gujska

**Affiliations:** 1 Department of Public Health, Medical University of Warsaw, 02-091 Warszawa, Poland; 2 Third Department of Psychiatry, Institute of Psychiatry and Neurology, 02-957 Warszawa, Poland; czajkowska.daria@gmail.com; 3 Masovian Specialized Health Center, 05-802 Pruscow, Poland; marcinkolodziej@yahoo.com; 4 Department of Psychology, SWPS University of Social Sciences and Humanities, 03-815 Warszawa, Poland; julkagujska@gmail.com

**Keywords:** anxiety, anxiolytic, cannabinoids, CBD, medical marijuana, psychiatry, psychosis, PTSD, THC, treatment

## Abstract

The rising popularity of medical marijuana and its potential therapeutic uses has resulted in passionate discussions that have mainly focused on its possible benefits and applications. Although the concept itself seems promising, the multitude of presented information has noticeable ramifications—terminological chaos being one. This work aimed to synthesize and critically analyze scientific evidence on the therapeutic uses of cannabinoids in the field of psychiatry. Emphasis was placed on the anxiolytic effects of cannabis constituents and their effects on post-traumatic stress disorder, anxiety disorders, schizophrenia spectrum, and other psychotic disorders. The review was carried out from an addictological perspective. A database search of interchangeably combined keywords resulted in the identification of subject-related records. The data were then analyzed in terms of relevance, contents, methodologies, and cited papers. The results were clear in supporting one common conclusion: while most findings provide support for beneficial applications of medical marijuana in psychiatry, no certain conclusions can be drawn until larger-scaled, more methodologically rigorous, and (preferably) controlled randomized trials verify these discoveries.

## 1. Introduction

Reports on the composition of so-called “medical marijuana” (MM) have been appearing in various forms of literature, whether scientific, popular science, or in the press. In consequence, not only are concepts being mixed and confused but a catalog of plausible indications outlining MM’s therapeutic potentials has even been created. This has resulted in complete chaos in terms of terminology. Interestingly, whenever a less enthusiastic or strikingly critical voice would appear on this matter, a reaction full of ostracism would follow. At present, a gradual and ongoing process of “accustoming society to marijuana” is unfolding. The circumstances surrounding this process are changing rapidly, and with more arising publications and emerging enthusiastic reports, the potential ramifications are becoming serious.

Discussions on potential applications of MM have been taking place in European countries, including Poland, for several years now. These debates show a clear polarization between admiration and condemnation of the substance. Unfortunately, they often fail to differentiate between whether the specimen’s discussed effects are the effects of dried cannabis inflorescences; its derivatives, such as oils or hashish; or cannabinoid-derived drugs.

Currently, about 150 million United States inhabitants have access to a product that in reality has little known but possibly some promising medicinal properties. The percentage of so-called MM users in the US has increased from 1.2% in 2013 to 1.6% in 2015, while an increased interest in the specimen has been noted in all states [1].

Interestingly, when analyzing and comparing the results of empirical research on alcohol and other psychoactive substances, one may observe that much remains unexplored in terms of cannabis. While the implications for cannabis’ therapeutic use in mental disorders require further investigation, some reports indicate that cannabis may be a beneficial additive or alternative to the treatment of PTSD and depression.

The following work emphasizes the systematization of knowledge in terms of reports outlining the influence of cannabinoid-derived drugs and dried cannabis inflorescences on the most frequently described mental disorders. The undertaken approach provides some novelty, due to the work’s design and methodology, which imply the necessity of verifying and examining the evidence that is recalled by sources, which serves as a basis for the currently postulated recommendations and applications in regards to MM. A review of the literature, research, and review papers was carried out from the standpoint of addictology, with due diligence to maintain objectivity. Review papers were analyzed in terms of their cited papers and conclusions drawn from them, while research was evaluated in terms of their methodologies and discussed conclusions.

### Ordering Terminology

The term “medical marijuana” (MM) does not exist in the statutory language; it is most commonly used in literature, mainly in journalism. Whenever referred to in the context of scientific publications, according to their contents, it should pertain to studies on the usage of burned, dried Cannabis sativa inflorescences. For the purpose of this text, the abbreviation MM will be used whenever “medical marijuana” is mentioned. In other instances, the features of the used specimens will be presented in detail.

Cannabis sativa extract consists of over 144 differing cannabinoids [2]. Currently, the two most frequently mentioned include cannabidiol (CBD), and delta-9-tetrahydrocannabinol (Δ9-THC), a cannabinoid with the greatest psychomimetic properties [2,3].

The most popular forms of marijuana such as dry cannabis and hashish, frequently used in the 1960s and 1970s, contained less than 4% of THC and often an equal proportion of “toning” CBD. However, in other countries, stronger varieties have been used, for example, a variety that is unable to produce high concentrations of both cannabinoids, the so-called Skunk, contains an average of 16% THC and a trace amount of CBD. Common in the Netherlands, Nederwiet contains up to 60% THC and is legally permitted for recreational use or fabrication of cannabis wax, which contains around 90% THC [4].

In recent years, synthetic cannabinoids, which are very popular among “designer drug” users, have been introduced to the market. Unlike THC, which is a partial agonist of the cannabinoid type 1 receptor (CB1R), most synthetic cannabinoids hold stronger psychomimetic properties due to being full agonists of CB1R [4].

## 2. Literature Review

Literature on “medical marijuana” (MM) and its potential therapeutic uses in the field of psychiatry has been analyzed for the purposes of this study. A search of databases, including PubMed, Cochrane Library, EvidenceAlerts, and Medscape, was conducted using different combinations of keywords such as “cannabinoids”, “marijuana”, “medical marijuana”, “THC”, “CBD”, “tetrahydrocannabinol”, “cannabidiol”, and “cannabinoid system”, interchangeably combined with the following: “treatment”, “therapeutic”, “psychiatry”, “psychosis”, “schizophrenia”, “anxiety”, “phobia”, “PTSD”, “mood disorders”, and “depression”. The search netted over 100,000 articles, published between the years 1970 and 2020, contents of which were then analyzed. Items that pertained to other concepts, mainly those concerning non-therapeutic matters or social analyses, were rejected. Given that publications no older than 10 years were those of focus, the final selection included the most recent reports. Some records were removed from the analysis due to their unavailability. The sources, which were identified with regards to depression and mood disorders, have been excluded from the final analysis as they have failed to meet the inclusion criteria. The credibility of indicated potential therapeutic applications was assessed by analyzing either the contents of referenced papers or the methodologies of research.

## 3. Results

Table A1 and Table A2 are attached to the Appendix A section of this paper and presents a descriptive synthesis of the most valuable quantitative and qualitative findings of both the review works and their cited contents, described in further detail below.

## 4. Discussion

### 4.1. The Neurobiology of Cannabis-Induced Psychoses

Cannabis can induce numerous complex neuropharmacological effects. They influence the limbic system and cognitive functions, among others. Tetrahydrocannabinol (THC) alters the response of neurons involved in working memory tasks [5,6] in a way that is similar to schizophrenia [7]. Experiencing psychosis is associated with having an altered perception of reality and a heightened sense of threat [8]. THC may have an anxiogenic effect, partially due to pathways bypassing the amygdala; moreover, it may affect areas that are connected with emotional perception. THC alters the amygdala’s response to the transmission of danger-sensation while also reducing the transmission of stimuli between the amygdala and the cerebral cortex [9]. The association between cannabinoid type 1 receptors (CB1R) midbrain participation and the feeling of a sense of threat was described in animal research models [10]. The study evaluated 358 rats of the same weight that were kept under similar conditions with controlled temperature and light. The rats were administered with i.a. N-methyl-D-aspartate (NMDA), cannabinoid type one (CB1), capsaicin, riluzole, and DL-2-amino-5-phosphonopentanoic acid (AP5). Their behavior was then observed for 4 days, during which researchers studied their reactions to odors of the above-mentioned substances. At the end of the experiment, the rats were anesthetized with isoflurane and sacrificed, with their brains being subjected to a histopathological examination. Results indicated that aversive learning relies on the fine-tuning of transient receptor potential vanilloid type 1 channel (TRPV1), CB1, α-amino-3-hydroxy-5-methyl-4- -isoxazolepropionic acid (AMPA) receptors (from pre- and postsynaptic membranes), and metabotropic glutamate, while NMDA receptors are responsible for immediate defensive responses. Dorsolateral periaqueductal activity seems to determine principal affective aspects of local TRPV1/CB1 balance-controlled aversive memory formation.

Structural changes that appear in connection with the early initiation and intensity of cannabis use include the hippocampus bilaterally [11,12] and the amygdala [13]. Simultaneously, such changes alter the connectivity between processing and executive systems [14,15,16,17], which reflects the concept of schizophrenia symptomatology. Heavy cannabis use has been linked to the dysfunction of the endocannabinoid system, CB1R in particular [18]. Differences in the availability of the CB1R were found between two groups of men, one consisting of males meeting the criteria for cannabinoid addiction (*n* = 11) and the other consisting of healthy subjects (*n* = 21). The structures in which significant differences (*p*-value < 0.01) were found included the amygdala, caudate, anterior and posterior cingulate cortex, frontal cortex, hippocampus, insula, occipital cortex, parietal cortex, putamen, and temporal cortex. The thalamus and the cerebellum constituted the only brain regions in which no significant differences were found. Adjusting for multiple comparisons indicated that the between-group dissimilarities found in the anterior cingulate cortex, frontal cortex, insula, hippocampus, parietal cortex, and temporal cortex remained significant. Trend level differences were identified in the amygdala and putamen.

The development of a condition that is clinically similar to schizophrenia, secondary to heavy cannabis use, may be caused by non-hyperdopaminergic processes [19], as opposed to endogenous schizophrenia [20]. Accordingly, it could be theorized that there exists a different mechanism of psychosis, connected with excitatory-inhibitory disorders of the GABA (Gamma-Aminobutyric Acid)-ergic [21] and glutamatergic systems [22], which are tightly modulated by the endocannabinoid system. Neurocognitive, neurochemical, and structural changes may lead to the development of clinical schizophrenia symptoms in individuals that are susceptible to the harmful effects of cannabis. This phenomenon can be explained by two theories: (1) cannabis use increases one’s susceptibility to psychosis; and (2) cannabis use results in the creation of additional pathways, which may lead to the onset of clinical symptoms of schizophrenia.

One of the first studies [23] that used neuroimaging techniques to investigate the interrelation between cannabis and psychosis utilized computed tomography (CT). The study consisted of 22 subjects, 12 with drug-induced paranoid hallucinatory states (using substances such as cannabis, opiates, LSD, cocaine, and other medications) and 10 with schizophrenia and no drug use. The study assessed CT images for macroscopic structural changes in the brain. No significant morphological differences were found between groups. Due to the study’s small sample size, no inferences could be drawn from the results.

Another study [24] assessed the cognitive functions and brain structures of patients who had experienced their first episode of psychosis and were marijuana users. Gray matter and lateral ventricular volumes were evaluated in 28 patients with the first episode of psychosis and a history of marijuana use, 78 patients with psychosis and no history of cannabis use, and 80 healthy individuals with no interview of marijuana use as the control group. Cognitive functions were rated on the Wechsler Memory Scale, 3rd edition (WMS-III), and the Controlled Oral Word Association Test (COWAT). Gray matter volume deficits (found in the medial temporal lobe and prefrontal cortex) typical of psychosis patients with no cannabis use history have not been confirmed in patients who have experienced the first episode of psychosis, secondary to cannabis use. Thus, the results suggest that a different neural pathway may be more involved in the development of psychosis in cannabis users than in non-cannabis users.

A study of small sample size [25] evaluated patients experiencing an acute psychotic episode within the course of schizophrenia, whether connected or not connected to cannabis use (*n* = 5 and *n* = 5, respectively) with recent-onset (<5 years) or substance-induced psychotic disorder (SIPD) (*n* = 6). All SIPD patients were abusers of cannabis. All patients were assessed with the use of PANSS (Positive and Negative Syndrome Scale), urine toxicology tests, and 2-deoxy-2-[fluorine-18]fluoro-D-glucose (18-FDG-PET) scans of the brain at resting state. Statistical analysis of variance (ANOVA) was performed with Statistical Parametric Mapping (SPM8) and the Scenium software. Upon comparison with the control group, the SIPD group was found to show bilateral hypermetabolism in the posterior cingulum and the precuneus: the two crucial default mode network regions of the human brain.

Another study assessed the brains of adolescent schizophrenia patients in terms of changes to their cognitive and structural functions (grey and white matter). The study consisted of 60 participants, 32 of which were adolescence-onset schizophrenia (AOS) patients. The remaining participants constituted the control group (*n* = 28). AOS patients were split into two equal groups (*n* = 16), one that was composed of CAN+ (cannabis positive: using cannabis more than three times a week, for at least 6 months) participants and the other consisting of CAN− (cannabis negative: not using cannabis) participants. The study used imaging methods, such as voxel-based morphometry (VBM) and diffusion tensor imaging (DTI) magnetic resonance imaging (MRI) techniques. Changes in grey matter density were found in the following areas: temporal fusiform gyrus, parahippocampal gyrus, ventral striatum, right middle temporal gyrus, insular cortex, precuneus, right paracingulate gyrus, dorsolateral prefrontal cortex, left postcentral gyrus, lateral occipital cortex, and cerebellum. No cognitive differences were found between the CAN− and CAN+ groups; the impairment was relative to that of controls [26].

### 4.2. Do Cannabinoids Cause or Heal Psychoses?

Reports on the influence of cannabis with respect to the occurrence of a schizophrenic episode, secondary to cannabis use, have emerged over 30 years ago [27]. Further reports and studies appear regularly [4]. Cannabinoid intoxication may lead to the development of temporary psychosis-like symptoms. The outlined psychotropic effects include depersonalization, derealization, racing thoughts, and disorganized thinking. Another possibility includes the emergence of visual and auditory hallucinations (the latter occurring less frequently) as well as delusions, including persecutory, sexual, and grandiose hallucinations. Psychotic symptoms may be observed in patients who have used cannabis with a high concentration of THC.

A study of 68 participants investigated the association between the age of onset of the first psychotic symptoms and the age of onset of cannabis use. The participants all showed at least one of the following: reduced functioning and familial risk or schizotypal personality; attenuated psychotic symptoms; brief, limited, or intermittent psychotic symptoms; or basic symptoms. It was found that more than half of first-psychosis symptoms occurred in conjunction with cannabis use. Further, it was found that the earlier cannabis use was initiated, the earlier the first cluster symptoms occurred (*p* = 0.001) [28]. Other review papers also emphasized that patients who develop psychosis after using cannabis usually experience an earlier onset of the disease and have better cognitive and social functions than other schizophrenia patients [29].

It is worth highlighting that careful monitoring of schizophrenia patients who use cannabis is key due to poorer prognosis, especially in cases of using strains that cause high psychotropic effects [30]. Numerous studies have examined the causal relationship between cannabis use and the onset of psychosis, paying special attention to THC intoxication [31,32,33,34,35,36,37], and long-term, intensive cannabis use [32,33,38]. Cannabis use has been associated with impaired attention in adolescents with early-onset schizophrenia [39]. Acute anxiety and delusional disorders are mentioned amongst complications of synthetic cannabinoid use. However, few substances have been tested experimentally (including animal experimentation) due to the fact that novel substances of this type are constantly emerging [40].

Review papers were systematized in terms of therapeutic uses of cannabinoids in the treatment of psychosis. The association between cannabinoids and the development of psychosis has been described in numerous reviews and several research papers. Most clinical trials included small sample sizes, loosely stringent inclusion and exclusion criteria, or simplified methodological protocols. In consequence, although promising according to their authors, the studies’ results cannot constitute a source of fundamental knowledge. Rather, they can indicate potential directions for further, in-depth, well-structured research [41].

Research is being conducted in order to evaluate the effects of both THC and cannabidiol (CBD). A study investigated acute psychotic episode patients (*n* = 11) who were orally administered with 10 mg of THC. The aim of the study was to assess the neural response to THC. The results were then compared with a similar-sized control group (*n* = 10) composed of people who had not experienced psychosis. The study’s small sample size was its main limitation; the results of the study were used to formulate certain observations regarding the different functioning of the brain’s selected areas, but the results do not allow for drawing convincing conclusions [42].

Some studies reported that CBD has a potentially beneficial, symptom-reducing effect on psychotic disorders [43,44], while others found CBD to have moderate-to-no reduction effects on psychotic experiences within acute psychosis [45,46,47].

Differences found between psychosis patients who use and do not use cannabis can potentially suggest a distinct subtype of schizophrenia that is secondary to intensive cannabis use (“secondary schizophrenia”) [22,23,24]. Both prophylaxis and treatment may be impacted by these findings, which still require further research in relation to the phenomenology of schizophrenia. However, understanding common mechanisms may bring novel treatment aims, which could prove to be revolutionary in the same manner as Kane’s pioneer work on clozapine, published over 30 years ago [48]. It is currently being postulated that CBD may hold antipsychotic properties that allow it to counteract psychotic symptoms and cognitive dysfunctions induced by Δ9-THC. This work, despite containing a summary of then-current review papers, cannot provide any justification or evidence for such a thesis. The authors themselves argue that due to legislative circumstances and the media’s influence on increasing cannabinoids’ “applicability’’ in medicine, conducting experimental and observational studies is necessary for providing conclusive evidence of their therapeutic efficacy. Nonetheless, CBD seems to be a more promising cannabinoid than THC in virtue of its potentially lower harmfulness [2].

### 4.3. Cannabis and Its Anxiolytic Properties

Animal and human studies suggest that CBD carries anxiolytic properties. The supposed anxiolytic effects occur due to its influence on receptors responsible for modulating behaviors attributed to fear and anxiety. The following constitute the aforementioned: cannabinoid type 1 receptor (CB1), transient receptor potential vanilloid type 1 channel- TRPV1 (originally called capsaicin, or vanilloid receptor VR1), and 5-hydroxytryptamine (serotonin) 1A receptor (5-HT1a) [49]. In some research papers, inferences were drawn from a small sample size.

A double-blind randomized controlled trial from 1982 aimed to verify if cannabidiol has the ability to reduce anxiety provoked by Δ9-THC in normal participants. Further, it aimed to study whether the effect appears due to a general block of the Δ9-THC action or a particular anxiolytic effect. The participants (*n* = 8) were aged between 20 and 38 years ($\bar{x}$ = 27) and included both males (*n* = 6) and females (*n* = 2). Some participants (*n* = 5) had smoked marijuana in the past, though not less than 15 days prior to the experiment commencing. All participants had a university education, were volunteers, and were in good mental and physical condition. The participants were administered with 0.5 mg/kg Δ9-THC, 1 mg/kg CBD, or a mixture containing 0.5 mg/kg Δ9-THC and I mg/kg CBD, and placebo or diazepam (10 mg). Each participant received the substances in a different sequence. The results suggested that CBD’s effects, contrary to those of Δ9-THC, are possibly connected to the antagonism of effects between the two cannabinoids. Further, it was found that the volunteers experienced a noticeable elevation in their anxiety level upon Δ9-THC ingestion [50].

Another randomized controlled trial aimed to test ipsapirone and cannabidiol’s acute effects in healthy participants subjected to a simulated public speaking (SPS) test and to compare them to anxiolytic effects of the benzodiazepine diazepam and placebo. The study was conducted following a double-blind procedure. The participants (*n* = 40) were healthy, aged between 20 and 30 years ($\bar{x}$ = 22.8), and included males (*n* = 18) and females (*n* = 22). All were recruited from voluntarily submitted psychology or medicine students, and compensation was given. The participants had to fit into pre-set criteria in order to qualify for the study. The studied subjects (*n* = 40) were randomly allocated into four equal groups (*n* = 10), where each group received one of the three mentioned substances, with the latter receiving a placebo. Each volunteer was subjected to only one experimental condition. Assessments were made with the use of the visual analog mood scale (VAMS), Spielberger state-trait anxiety inventory (STAI), bodily symptoms scale (BSS), and digital-symbol substitution test (DSST). Physiological measures such as blood pressure and heart rate were also noted. The results confirmed that the SPS test is sensitive to drug effects and induces reliable increases in anxiety. The VAMS scale results showed that ipsapirone attenuated SPS-induced anxiety, while CBD reduced post-SPS test anxiety. Diazepam held anxiolytic properties both before and after the SPS test and had no effect on the increase of SPS test-induced anxiety. Only ipsapirone attenuated the systolic blood pressure, which was SPS test-induced. Significant sedative effects were only seen with diazepam. The findings suggest that CBD, as well as ipsapirone, may hold anxiolytic properties (as shown by its effects) when acutely administered to healthy individuals subjected to stressful situations [51].

Fusar-Poli et al. aimed to research the effects of Δ9-THC and CBD on regional brain function during emotional processing in a double-blind randomized controlled trial, published in 2009. The study involved healthy English-native, right-handed, male subjects (*n* = 15), aged between 18 and 35 years ($\bar{x}$ = 26.67; σ = 5.7). The participants were recruited through an advertisement in the local media. Their IQ ($\bar{x}$ = 98.67; σ = 7.0) was measured using the National Adult Reading Test, while cannabis and other illicit substance use were determined with the use of the Addiction Severity Index. No participant had used cannabis in the last month nor had a family history of mental illness, alcohol or other drug abuse, or dependence. All were asked to follow specific instructions for substance abstinence. MRI scans and skin conductance responses (SCRs) were taken at three different time points. In terms of psychopathological measures, data were taken periodically with the use of the VAMS, STAI, Analogue Intoxication Scale (AIS), and PANSS. The procedure also included monitoring the blood pressure and heart rate, and blood samples were taken. Distinct modulatory effects of cannabidiol and Δ9-THC were found in terms of the regional neural response to fearful faces. When exposed to fearful stimuli, cannabidiol reduced the neurofunctional engagement of the amygdala and cingulate cortex, which was found to be correlated with a decrease in the electrodermal response, consistent with its reported anxiolytic effects. Δ9-THC was related to an increase in anxiety and electrodermal response. It also modulated activation in parietal and frontal areas. Overall, the results showed that Δ9-THC increased anxiety, intoxication, sedation, and psychotic symptoms, while CBD was associated with an anxiety symptom-reducing tendency [52].

Anxiolytic properties of cannabis were also tested on patients with Social Anxiety Disorder (SAD), also referred to as social phobia. In 2011, Crippa et al. conducted a double-blind randomized controlled trial, which aimed to assess the anxiolytic effect of CBD in social phobia patients, with the use of functional neuroimaging. Another goal was to inquire whether a possible anxiolytic effect of CBD on social phobia patients would be related to the modulation of the functional activity of temporo-limbic structures and paralimbic regions. The participants were recruited from an epidemiological sample of university students; they were required to fit into pre-specified criteria in order to qualify. The studied sample (*n* = 10) consisted of male, right-handed treatment-naïve social phobia patients aged between 20 and 33 years ($\bar{x}$ = 24.2, σ = 3.7). The disorder severity was assessed with the use of the Brief Social Phobia Scale (BSPS) and the Social Phobia Inventory (SPIN). All participants were classified as suffering from severe social phobia. In one session, the subjects were administered either 400 mg of CBD or a placebo, while in the other session, they received the drug that was not administered in the prior one. The subjects were asked to rate the severity of their anxiety via the VAMS. Measurements of their regional cerebral blood flow were taken with the use of 99mTc-ECD brain perfusion, single-photon emission computerized tomography (SPECT). Compared to placebo, the administration of CBD was associated with a decrease in subjective anxiety and ECD uptake of the left parathyroid gyrus, hippocampus, and temporal gyrus, as well as an increase in ECD escapement of the right bend of the rim. These findings suggest that in the case of social phobia patients, acute CBD administration has the potential to reduce anxiety, possibly due to its altering effect on the functional activity of brain areas involved in anxiety processing. According to the authors, results suggest that CBD reduces anxiety in social phobia because of its effects on the limbic and paralimbic areas of the brain [53].

A double-blind randomized controlled trial from 2011, done by Bergamaschi et al., aimed to study the effects of CBD or placebo on a simulation public speaking test (SPST) in healthy control patients and treatment-naïve social phobia patients. The study enrolled participants (*n* = 36) based on a screening procedure, which consisted of self-assessment using the short version (MINI-SPIN) of the SPIN. Diagnoses were further confirmed with the use of the Structured Clinical Interview for the DSM-IV (Diagnostic and Statistical Manual of Mental Disorders 4th edition), clinical version (SCID-CV). Patients with social phobia (*n* = 24) were split into two equal groups (*n* = 12), with one receiving 600 mg of CBD and the other receiving placebo. Healthy control patients (*n* = 12) received no medications. Psychological assessments were made with the use of the VAMS, the Negative Self-Statements Public Speaking Scale (SPSS-N), and the BSS. In terms of the physiological measures, skin conductance was measured with a computer-controlled, voltage-constant (0.6 V) module with an automatic back off, arterial blood pressure was measured with a mercury sphygmomanometer, and heart rate was estimated based on a manually counted pulse rate. In terms of the VAMS, results showed no significant differences regarding cognitive impairment, discomfort, and alert factors between CBD and placebo receiving groups. However, an increase in negative self-evaluations was found in the placebo group, while in the CBD group, it was almost abolished. In comparison to the control group, the placebo group presented significantly higher anxiety along with greater cognitive impairment, discomfort, and alertness. Pretreatment with CBD was found to significantly reduce anxiety, cognitive impairment, and discomfort in social phobia patients’ speech performance in a way that matched the healthy control group. The same improvement was noticed in terms of alertness in their anticipatory speech. These findings suggest that in social phobia patients, the anxiety-enhancing effects of SPST may be reduced by CBD. Further, they indicate that the cannabinoid inhibits one of the main symptoms of the disorder: fear of public speaking. More research is needed to verify whether such conclusions can be drawn [54].

Other papers with worthy findings included literature or systematic reviews. Research reviews and conclusions drawn from them are similar to the ones presented above [55,56,57]. One review found further support for cannabinoids’ positive effects on reducing anxiety. The findings suggested that THC has the capability of reversing anxiety-like behavior, given that it is one of the CB1R agonists. Moreover, recreational marijuana use was frequently associated with anxiety reduction. It was determined that most chronic cannabis users engage in this behavior due to cannabis’ anxiolytic and stress-reducing properties [58,59]. Another review work analyzed evidence that indicated a therapeutic role of CBD in the treatment of fear, anxiety, or any trauma-related condition. The control trial findings seemed promising, though the authors concluded that because of insufficient proof, it is impossible to state for certain that CBD is an effective treatment for mood, sleep, and anxiety complaints [60]. One conclusion on cannabis and its anxiolytic properties that seems to be presented in the analyzed systematic review [49,61] and literature review papers is that, despite possibly-promising reports, more methodologically-valid and representative-based evidence is warranted for any firm conclusions to be drawn. Finally, ongoing research is taking place. Van der Flier (2019) has published a protocol for a randomized controlled study evaluating CBD’s effect (as an adjunct to exposure therapy) on reducing phobia symptoms (social phobia or panic attacks with agoraphobia), though the results have not been published as of the present.

### 4.4. Cannabis and Post-Traumatic Stress Disorder

There is a need for novel profile-varied pharmacological-treatment options in the treatment of post-traumatic stress disorder (PTSD), facilitating the efficacy and specificity of symptom targeting. Imaging evidence suggests that the endocannabinoid system may be involved in PTSD pathophysiology, with a lower endocannabinoid tone found in the amygdala-hippocampal-cortico-striatal circuitry [62]. Accordingly, Hill et al. theorized that endocannabinoid deficiency may lead to increased stress susceptibility and psychopathological predisposition, which would, in turn, facilitate trauma development. Such a relation could provide further insight into the biological basis of cannabinoids’ popularity among patients with PTSD [58]. Furthermore, animal research on mice subjected to traumatic shock treatments revealed that upon endogenous cannabinoid deficiency correction, the mice were able to defeat their conditioned response due to the inhibition of g-aminobutyric acid pathways in the amygdala, allowing for the elimination of harmful, stress-inducing memories. Interestingly, the same mechanism is thought to account for human responses to cannabinoids [63].

In 2009, an open-label clinical trial by Fraser et al. aimed to investigate the effects of nabilone as an adjuvant to standard pharmacotherapeutic treatment in patients experiencing non-treatment responsive nightmares within the course of PTSD. The study included male and female civilians (*n* = 47), diagnosed with PTSD, and experiencing PTSD-related nightmares for the duration of at least two years, with a frequency of at least one nightmare per week. All patients were receiving psychotropic medications for PTSD, thereby allowing for the assessment of nabilone use as an adjuvant to standard treatment. The measures included self-report in the form of a sleep and nightmare tracking sheet, commencing a week prior to starting nabilone and weekly thereafter, concluding when met with satisfactory trial results. In any case of side effect occurrence, the trial was terminated. Nabilone was reported to either terminate nightmares completely or reduce the nightmare intensity in a significant manner. However, thirteen patients discontinued the nabilone therapy due to moderate-to mild side effects. Some patients also reported improvements in their sleep quality. These findings suggest that nabilone may be an appreciable treatment for PTSD-related nightmares. Nonetheless, the authors emphasize the importance of conducting further, more sizable randomized controlled trials, testing the effects of nabilone on the whole spectrum of PTSD symptoms. [64]

A retrospective chart review study by Greer et al. from 2015 aimed to study and analyze data on symptoms of PTSD, acquired in psychiatric evaluations of the New Mexico Medical Cannabis Program patients. The PTSD patients (*n* = 80) had to fit into specific criteria, including being classified as meeting DSM-IV criterion A for PTSD; reporting the presence of criteria B, C, and D symptoms when not using cannabis; feeling significant symptom relief when using cannabis; and lacking any harms or problems in relation to functioning when using cannabis. The measures included retrospective administration of the Clinician-Administered Posttraumatic Scale for DSM-IV (CAPS). The results showed a 75% CAPS symptom score reduction when patients used cannabis, compared to when they did not. Thus, in some patients, an association was found between cannabis and PTSD symptom reduction. However, prospective, placebo-controlled research is required in order to give further support to the efficacy of cannabis and its components in PTSD treatment. [65]

An open-label trial by Roitman et al. in 2014 aimed to explore the tolerance, safety, and preliminary clinical effects of Δ9-THC as an adjuvant to standard therapy in patients with unremitting chronic PTSD. The study included outpatients recruited from mental health clinics in Jerusalem, Israel. The subjects were diagnosed with chronic PTSD, for which they were receiving psychopharmacological treatment (*n* = 10), with no cannabis use in the last six months. The physiological measures included assessments of blood pressure, heart rate, and body mass index (BMI). Many psychometric instruments, including CAPS, clinical global impression scale (CGI), Pittsburgh Sleep Quality Index (PSQI) and its PTSD addendum, Nightmare Frequency Questionnaire (NFQ), and Nightmare Effects Survey (NES) were used. The administration took place twice a day. The starting dose was set at 2.5 mg of THC THC twice a day, meaning that the daily dose amounted to 5 mg of THC. If well-tolerated, the dose was increased to 5 mg of THC twice a day, equaling 10 mg of THC per day. The trial lasted for a period of three weeks. In the end, all patients received the maximal, increased dose of THC. There were mild adverse effects in four patients, none of which led to treatment discontinuation. The intervention resulted in a statistically significant decrease in symptom severity, as observed in CGI-S (CGI-Severity), CGI-I (CGI-Improvement), total NES scores, sleep quality, frequency of nightmares, and PTSD hyperarousal symptoms. The results suggest that orally absorbable Δ9-THC is safe and well-tolerated by patients with chronic PTSD. Greater motivation in terms of using cannabis for sleep purposes was reported by PTSD, rather than non-PTSD, patients. A possible explanation of nightmare reduction and sleep quality improvement is that THC was found to have a modifying effect on sleep architecture. Specifically, Δ9-THC seemed to deplete the REM (rapid eye movement) phase of sleep (in which nightmares occur) and enhance non-REM phase 4 sleep (the restoring phase of sleep). However, more randomized controlled trials, preferably placebo-controlled, are needed in order to confirm the findings [66].

A retrospective study by Cameron et al., from 2014, aimed to investigate the effects of Nabilone in relation to various aspects including but not limited to PTSD-related nightmares, chronic pain, and harm reductions. The subjects were recruited from the secure treatment unit (STU) in the St Lawrence Valley Correctional and Treatment Center. The study consisted of male prisoners (*n* = 104) who had been clinically diagnosed with severe mental illnesses and were not using Nabilone at the time of admission. Their ages ranged between 19 and 55 ($\bar{x}$ = 32.7) years. The measures included the following self-reports: the Posttraumatic Checklist Civilian version C (PCL-C) and Global Assessment of Functioning (GAF). All measures were repeated pretreatment and posttreatment. In the case of self-reports on sleep and nightmares, pretreatment measures were taken a week prior to nabilone initiation and posttreatment measures were taken a week post-final nabilone administration, respectively. GAF and PCL-C scores were taken at the time of admission (as pretreatment measures) and at the time of discharge (as post-treatment measures). The final dose of nabilone was averaged at 4.0 mg. All pretreatment and posttreatment measures signified remarkable progressions. The majority of patients experienced either a significant decrease in nightmare intensity or cessation of nightmares. There was a significant reduction in PCL-C scores, congruent with a reduction in PTSD symptoms. A decrease in functioning impairments was observed in the GAF scores, which increased significantly. These findings imply that patients with PTSD, in whom nightmares persist despite standard pharmacotherapy, could benefit from the synthetic cannabinoid- nabilone. The limitations of this study were mainly due to the retrospective design of the study and self-report nature of measurements, lack of control group, and concurrent pharmacological (psychotropic medications) and psychotherapeutic treatments. The authors indicated future directions for research, including a randomized controlled trial on the comparison of nabilone with placebo and prazosin in PTSD-related insomnia and nightmares as well as the effects of the three substances on other PTSD symptoms. Additionally, it is important to mention that nabilone should be studied in terms of harm reduction [67].

Lastly, in 2015, Jetly et al. conducted a preliminary, randomized, double-blind, placebo-controlled, crossover design study. The aim of the study was to assess whether nabilone capsules are effective in terms of reducing the intensity and frequency of PTSD-related nightmares. The sample consisted of currently-serving military men (*n* = 10) diagnosed with PTSD and experiencing nightmares despite participating in standard treatment. The participants were subjected to a physical exam, based on which they were either included or excluded from the sample. In terms of the final sample, they were instructed to continue pharmacotherapy or psychotherapy if they had done so at the time of study entry. The measures included self-report, such as selected CAPS items, the CGI-C (Clinical Global Impression of Change), the PTSD dream rating scale, and the WBQ (General Well Being Questionnaire), administered at the commencement and completion of each trial period. Another self-report measure, a sleep diary log, was completed within the final week of each of the trial periods. The subjects were observed during two periods, each lasting 7 weeks, split by a 2-week wash-out period. Two equally-sized groups were created: the patient’s vital signs and nightmare-related experiences were screened weekly. The patients were administered either 0.5 mg of nabilone or a placebo in the first study period, followed by the non-previously administered substance in the second period. The nabilone dose was titrated to an effective amount, though not exceeding 3 mg; maximum dosage was achieved by week 5, allowing for the investigation of its effects in the remaining duration of the study period (2 weeks). The results showed that nabilone brought significant relief to PTSD military personnel who did not respond to traditional therapies, suggesting that it is a clinically-relevant treatment alternative. However, it is very important for these results to be replicated in a more sizable sample. The authors also emphasized the need for research on nabilone’s effect on other PTSD symptoms, such as re-experiencing, insomnia, and hypervigilance [62].

Other systematic reviews and review papers analyzed the same papers as recalled above [55,58,68,69], with mentions of additional research. One work, evaluating animal studies on rats, stated that anxiogenic effects of PTSD may be lowered via cannabinoid treatment [69]. Some findings were contradictory to the reported benefits, suggesting that precursory heavy cannabis use may lead to lower treatment responsiveness. It was also reported that post-treatment cannabis initiation may lead to an increase in PTSD symptoms [68]. A systematic review and meta-analysis on cannabis medical uses across a wide range of conditions found that cannabinoids were associated with an elevated risk of short-term adverse effects [70]. However, collectively, these papers emphasized that although evidence seems to indicate that cannabis could be beneficial in palliating and decreasing symptoms of PTSD (such as hyperarousal, sleep, and nightmares), further research is required. The main indication for further research is in relation to limitations such as scanty, non-representative sample sizes, inaccurately rigorous methodologies, and follow-up deficiencies of present papers.

## 5. Conclusions

“Medical marijuana” (MM) is a broad term specifying a range of products, from whole-plant cannabis and its derivatives to synthetic cannabinoids. The two main constituents of the cannabis specimen, delta-9-tetrahydrocannabinol (Δ9-THC) and cannabidiol (CBD), have been reported to hold medicinal properties and produce beneficial therapeutic effects, while further favorable evidence has also been reported with regard to synthetic cannabinoids’ (ex. nabilone) medicinal uses. However, the multitude of information surrounding these concepts seems to lack order and empirical basis, introducing chaos and promoting exposure with little to no knowledge of their possible ramifications. The gathered literature pertains to the applications of MM in the field of psychiatry, specifically in relation to post-traumatic stress disorder, anxiety disorders, the schizophrenia spectrum, and other psychotic disorders. The analyzed papers often speculated that cannabinoids may provide promising treatment alternatives for these conditions. Although the idea of novel, propitious pharmacotherapeutic options could prove advantageous in terms of public health interest, the presented evidence-based inferences, which speak to the medical applicability of the cannabinoids, appear to lack solid, reliable evidence. As disclosed throughout the review, most papers, despite providing favorable findings to the medicinal uses of cannabis, cannot attest to the safety, tolerability, indications, and possible risks of its utilization. Multiple design drawbacks, which prohibit deducing certain conclusions on this topic, appeared in connection to scanty, non-representative sample sizes, inaccurately rigorous methodologies, and follow-up deficiencies. In accordance with the identified limitations, a large portion of the review papers emphasized the need for novel clinical trials of randomized, controlled, and preferably blinded nature. Therefore, if any conclusions were to be drawn from this literature review, let them be that (1) cannabinoids appear to hold beneficial medicinal properties; and (2) the medical uses of cannabinoids remain largely unexplored due to lacking valid, reliable, empirical, evidence.

## Data Availability

Not applicable.

## Abbreviations

**AIS**	Analogue Intoxication Scale
**AMPA**	α-amino-3-hydroxy-5-methyl-4-isoxazolepropionic acid
**ANOVA**	Analysis of Variance
**AP5**	DL-2-amino-5-phosphonopentanoic acid
**AOS**	Adolescence-onset schizophrenia
**BMI**	Body Mass Index
**BSPS**	Brief Social Phobia Scale
**BSS**	Bodily Symptoms Scale
**CAPS**	Clinician-Administered Posttraumatic Scale
**CBD**	Cannabidiol
**CB1R**	Cannabinoid type 1 receptor
**CGI**	Clinical Global Impression
**CGI-C**	Clinical Global Impression of Change
**CGI-I**	Clinical Global Impression of Improvement
**CGI-S**	Clinical Global Impression of Severity
**COWAT**	Controlled Oral Word Association Test
**CT**	Computed Tomography
**DSM-IV**	Diagnostic and Statistical Manual of Mental Disorders
**DSST**	Digit-Symbol Substitution Test
**DTI**	Diffusion Tensor Imaging
**GABA**	Gamma-Aminobutyric Acid
**GAF**	Global Assessment of Functioning
**LSD**	Lysergic Acid Diethylamide
**MINI-SPIN**	Mini Social Phobia Inventory
**MM**	Medical Marijuana
**MRI**	Magnetic Resonance Imaging
**NES**	Nightmare Effects Survey
**NFQ**	Nightmare Frequency Questionnaire
**PTSD**	Post-Traumatic Stress Disorder
**REM**	Rapid Eye Movement
**SAD**	Social Anxiety Disorder
**SCID-CV**	Structured Clinical Interview for DSM-IV, Clinical Version
**SCR**	Skin Conductance Response
**SIPD**	Substance-induced psychotic disorder
**SPECT**	Single-Photon Emission Computerized Tomography
**SPIN**	Social Phobia Inventory
**SPM8**	Statistical Parametric Mapping
**SPS**	Simulated Public Speaking
**SPSS-N**	Negative Self-Statements Public Speaking Scale
**SPST**	Simulation Public Speaking Test
**STAI**	Spielberger State-Trait Anxiety Inventory
**STU**	Secure Treatment Unit
**THC**	Tetrahydrocannabinol
**TRPV1**	Transient Receptor Potential Vanilloid type 1 channel
**US**	United States
**VAMS**	Visual Analog Mood Scale
**VBM**	Voxel-based morphometry
**WBQ**	General Well Being Questionnaire
**WMS-III**	Wechsler Memory Scale (3rd edition)
**5-HT1a**	5-hydroxytryptamine (serotonin) 1A receptor
**18-FDG-PET**	18-Fluoro-2-deoxyglucose Positron Emission Tomography
**Δ9-THC**	Delta-9-tetrahydrocannabinol

## Appendix A

**Table A1:** Descriptive Synthesis of the Analyzed Quantitative Review and Cited Works: Anxiety, Social Phobia, and Post-Traumatic Stress Disorder.

Authors and Date	Study Type and Research Design	Sample Characteristics	Cannabis Exposure	Experimental and Control Intervention	Outcomes
Zuardi AW, Shirakawa I, Finkelfarb E, and Karniol IG. (1982)	Quantitative Double-blind randomized controlled trial	*n* = 8 Male (*n* = 6) Female (*n* = 2) Aged 20–30 years ($\bar{x}$ = 27) Volunteers	Marijuana use (*n* = 5) no less than 15 days prior to the experiment	A mixture of 0.5 mg/kg Δ9-THC and 1 mg/kg CBD A mixture of 0.5 mg/kg Δ9-THC and I mg/kg CBD Placebo Diazepam, 10 mg	Δ9-THC’s anxiogenic properties led to a substantial elevation of anxiety, which was partially antagonized by CBD Subjective alterations provoked by Δ9-THC diminished with simultaneous CBD administration CBD’s effects seem to be connected to the antagonism of effects between the two cannabinoids
A.W. Zuardi, R.A. Cosme, F.G. Graeff, and F.S. Guimarães (1993)	Quantitative Double-blind randomized controlled trial	*n* = 40 Male (*n* = 18) Female (*n* = 22) Aged 20–30 years ($\bar{x}$ = 22.8) Paid volunteers, recruited from university students of Medicine and psychology courses	Not specified	Identical gelatin capsules with: CBD, 300 mg Diazepam, 10 mg Ipsapirone, 5 mg Placebo	Ipsapirone attenuated SPS-induced anxiety (and systolic blood pressure), while CBD reduced anxiety experienced after the SPS test Diazepam held significant sedative and anxiolytic effects and had no effect on the increase of SPS test-induced anxiety The SPS test is sensitive to drug effects and induces reliable increases in anxiety
Fusar-Poli P, Crippa JA, and Bhattacharyya S, et al. (2009)	Quantitative Double-blind, randomized, and placebo-controlled design	*n* = 15 English-native males Aged 18–35 ($\bar{x}$ = 26.67, SD = 5.7) Recruitment strategy not specified.	No cannabis use in the last month. Lifetime exposure of <15 times.	Gelatin capsules containing: Δ9-THC, 10 mg CBD, 600 mg Placebo	Cannabidiol reduced the neurofunctional engagement of the amygdala and the cingulate cortex at fearful stimuli exposure, which was correlated with a decrease in the electrodermal response, consistent with its reported anxiolytic effects Δ9-THC was associated with an increase in anxiety and electrodermal response. It also modulated activation in parietal and frontal areas
Fraser GA. (2009)	Quantitative Open-label clinical trial design	*n* = 47 Male (*n* = 20) Female (*n* = 27) Aged 26–68 ($\bar{x}$ = 44, SD = 9) Patients referred to a psychiatric specialist outpatient clinic by other physicians	Screened for previous negative experiences with marijuana use	Average effective dose of Nabilone = 0.5 mg (range: 0.2–4.0 mg) No control	The majority of patients experienced either a cessation or significant reduction in nightmare intensity Some reported an improvement in sleep quality and time, and a reduction in night sweats and daytime flashbacks Nabilone proved to be beneficial in patients with treatment-naïve nightmares, within the course of PTSD
Jetly R, Heber A, Fraser G, and Boisvert D. (2014)	Quantitative Preliminary randomized, double-blind placebo-controlled cross-over design	*n* = 10 Male Aged 18–65 ($\bar{x}$ = 43.6, SD = 8.2) Patients referred to a military treatment clinic	No illicit substance use	Starting dose: Nabilone, 0.5 mg Titrated to an effective, maximum dose of: Nabilone, 3.0 mg Placebo	Nabilone produced large or significant improvements in 70% of the patients Nabilone significantly reduced the frequency and intensity of nightmares Nabilone was well-tolerated by the patients
Roitman P, Mechoulam R, Cooper-Kazaz R, and Shalev A. (2014)	Quantitative Preliminary, open-label pilot study design	*n* = 10 Male (*n* = 7), Female (*n* = 3) Age ($\bar{x}$ = 52.3, SD = 8.3) Recruited from mental health clinics in Jerusalem, Israel	No cannabis use at least 6 months before the study	Starting dose: THC, 2.5 mg twice/day Final dose (if increased): THC, 5.0 mg twice/day No control	Significant improvements in sleep quality, nightmare frequency, and PTSD hyperarousal symptoms 20% of patients attained total nightmare remission Orally absorbable Δ (9)-THC was safe and well tolerated by patients with chronic PTSD
Crippa JA, Derenusson GN, Ferrari TB, et al. (2010)	Quantitative Double-blind placebo-controlled design	*n* = 10 Male Aged 20–33 years ($\bar{x}$ = 24.2, SD = 3.7) Recruited from an epidemiological sample of 2320 university students, selected via a screening procedure	Lifetime exposure of <5 times No marijuana use in the year prior to the study No illegal drug use	Gelatin capsules containing: CBD, 400 mg Placebo	Acute CBD administration has the potential to reduce subjective anxiety in SAD patients, possibly due to CBD’s altering effect on the functional activity of brain areas involved in anxiety processing CBD, relative to placebo, led to significant decrements in the parahippocampal activity The anxiolytic effects of CBD were not attributable to sedation
Bergamaschi MM, Queiroz RH, Chagas MH, et al. (2011)	Quantitative Double-blind randomized placebo-controlled trial	*n* = 36 SAD patients (*n* = 24) Healthy control group (*n* = 12) Recruited from 2319 undergraduate students, screened for probable SAD Groups matched according to sex, age, years of education, and socioeconomic status	Lifetime exposure of <5 times No marijuana use in the year prior to the study No illegal drug use	CBD gelatin capsules, 600 mg Placebo gelatin capsules No medications	CBD inhibits one of the main symptoms of SAD-speaking in public CBD pretreatment significantly reduced the anxiety, cognitive impairment, and discomfort in SAD patients’ speech performance and alert in their anticipatory speech An increase in negative self-evaluations presented by the placebo group (significantly greater cognitive impairment, higher anxiety, alert, and discomfort) was almost abolished in the CBD group

**Table A2:** Descriptive Synthesis of the Analyzed Systematic Review and Meta-Analysis Works.

Authors and Date	Aim	Eligibility: Inclusion and Exclusion Criteria	Analysis and Data Extraction	Results	Conclusions
Khoury et al. (2017)	The aim was to assess the use of CBD in the treatment of anxiety disorders, schizophrenia, psychotic disorders, bipolar disorder, depression, and substance use disorders. Emphasis was put on exploring the benefits and adverse events of cannabidiol’s applications in the aforementioned psychiatric conditions.	Assessment of CBD’s therapeutic use in the treatment of anxiety, psychosis, schizophrenia, depressive disorder, or substance use disorders. All types of study designs were included. Pre-clinical studies, expert opinions, literature reviews, research not pertaining to psychiatric disorders of interest, and duplicates were excluded from the analysis.	World Federation of Societies of Biological Psychiatry (WFSBP) guidelines were followed throughout the classification process. The Specific data sections, including references, study design, participant characteristics, primary goals, sample size, intervention type, results, and main limitations were extracted from the studies whenever possible.	Identification of 596 papers and 104 registered clinical trials that included CBD as a treatment strategy. 34 records included in the final analysis: Registered clinical trials: (*n* = 21) Articles: (*n* = 13)	Evidence on the use of CBD in acute anxiety and long-term SAD treatment was derived from uncontrolled studied, which lacked support. No identified studies assessing the impact of CBD on other anxiety disorders. Evidence for the short-term treatment of treatment resistant schizophrenia (TRS) arose from uncontrolled studies and lacked evidence. Negative evidence was found for first episode of schizophrenia. Evidence on the use of CBD in Cannabis dependence came from case reports and lacked evidence. Scarce evidence exists on the safety as well as the efficacy of CBD in the field of psychiatry. Well-designed, substantially larger RCT’s are crucial in order to assess the effects of CBD in psychiatric disorders.
Betthauser K, Pilz J, Vollmer LE. (2015)	The objective was to review the existing data on the efficacy, safety, and tolerability of cannabinoids in military veterans with PTSD.	Cannabinoids’ general use in persons with a PTSD diagnosis or cannabinoids’ use in the amelioration of PTSD symptoms, both in relation to military experience. Research in humans. English language. Subjects with diagnosed PTSD via a standard scale (ex. DSM-IV, or DSM-V). Editorials and opinion pieces were excluded.	Each item was analyzed both individually and collaboratively by the authors so as to establish its clinical relevance.	59 articles were identified via the database search. 11 articles were included in the final review. A variety of study designs were included in the final selection.	Further research in regards to cannabinoid’s therapeutic effects on PTSD symptoms is needed. The identified evidence mainly lacked randomization, a representative and sizeable sample, and placebo control. The assessed evidence suggests that some military veterans with PTSD use cannabis or its derivatives in order to control their PTSD symptoms. Some patients report benefits, such as reduced anxiety, insomnia, and improved coping ability. Further inquiry is much needed in order to get a better understanding of the phenomenon. In general, the articles supported two concepts: 1. Cannabis is used by persons with PTSD for symptom alleviation. 2. Some find cannabis to be beneficial in that sense.
Lim K, See YM, Lee J. (2017)	The aim was to provide a more extensive evaluation of the efficacy regarding medical uses of cannabinoids, specifically with regard to psychiatric, movement, and neurogenerative disorders.	RCT’s that compared and examined cannabis (as a pharmacological intervention) with placebo, usual care, cannabis derivatives, or other active treatments. Human studies on subjects of any sex and age, clinically diagnosed with: movement disorders, neurological conditions and psychiatric conditions. English language. Quantitative studies, as well as opinion and discussion papers, were excluded.	Data on the study type, sample profile, intervention dosage and type, primary outcome measures, and side effects and adverse events were extracted from each report. Methodological validity was assessed by two independent raters with the use of the Cochrane risk of bias tool. Any discrepancies were resolved through a discussion.	931 hits were originally identified. 24 records were included in the final review: Crossover trials (*n* = 18) Parallel trials (*n* = 6) The final study selection consisted of studies conducted in Western societies.	Although some trials reported positive findings in relation to anorexia nervosa, anxiety, PTSD, psychotic symptoms, agitation in Alzheimer’s disease and dementia, Huntington’s disease, and Tourette syndrome, and dyskinesia in Parkinson’s disease, a certain conclusion cannot be drawn from them. The evaluation of the trials’ resulted in an unclear risk of bias. It also indicated methodological issues such as inadequate descriptions of allocation concealment, blinding, and small sample sizes. More methodologically valid controlled trials are needed in order to assess both the long-term and short-term efficacy, tolerability, and safety of cannabis use in medicine along with the mechanisms underlying its therapeutic potential.
Whiting PF, Wolff RF, Deshpande S, et al. (2015)	The objective was to systematically review the benefits and adverse effects of cannabinoids’ use in the field of medicine.	RCT’s comparing cannabinoids with placebo, usual care or no treatment in nausea and vomiting due to chemotherapy, appetite stimulation in HIV/AIDS, chronic pain, spasticity due to multiple sclerosis (MS) or paraplegia, depression, anxiety disorder, sleep disorder, psychosis, intra- ocular pressure in glaucoma, or Tourette syndrome. No language restriction. Nonrandomized studies, including uncontrolled studies, in which more than 25 patients were eligible.	The data extraction was done by two independent reviewers and involved: categorical and continuous data, baseline characteristics and outcomes, reported between-group statistical analyses, and full contents. The study quality was assessed with the Cochrane risk of bias tool. Data were pooled using random-effects-meta-analysis if possible. Dichotomous data: odds ratio (OR) and confidence interval (CI) measures. The focus was on peer-reviewed articles. Synthesis analyses.	23,754 hits were originally identified. A total of 79 studies, available as 151 reports, were included in the final review: Parallel trials: (*n* = 34) Cross-over trials: (*n* = 45) Publication date range: 1975–2015 (ME = 2004). Trials: 5%: low risk of bias 70%: high risk of bias 25% unclear risk of bias	In terms of anxiety disorders, only one small parallel-group trial was judged to be at high risk of bias was identified. Other reports pertaining to anxiety in patients with chronic pain reported a larger benefit to cannabinoid use rather than placebo, but the studies were not restricted to anxiety disorder patients. Two studies were identified in relation to psychosis and judged at high risk of bias. No differences in mental health outcomes were found between treatment groups. In terms of improvements in nausea and vomiting due to chemotherapy, weight gain in HIV infection, sleep disorders, and Tourette’s syndrome, only low-quality evidence was found. An association was found between Cannabinoids and increased risk of short-term adverse effects (including serious adverse effects). The most commonly occurring adverse effects included dizziness, dry mouth, nausea, fatigue, somnolence, euphoria, vomiting, disorientation, drowsiness, confusion, loss of balance, and hallucinations.
